# Characterization of a New Cold-Adapted and Salt-Activated Polysaccharide Lyase Family 7 Alginate Lyase from *Pseudoalteromonas* sp. SM0524

**DOI:** 10.3389/fmicb.2016.01120

**Published:** 2016-07-19

**Authors:** Xiu-Lan Chen, Sheng Dong, Fei Xu, Fang Dong, Ping-Yi Li, Xi-Ying Zhang, Bai-Cheng Zhou, Yu-Zhong Zhang, Bin-Bin Xie

**Affiliations:** ^1^State Key Laboratory of Microbial Technology, Shandong UniversityJinan, China; ^2^Marine Biotechnology Research Center, Shandong UniversityJinan, China; ^3^Institute of Marine Science and Technology, Shandong UniversityJinan, China; ^4^Laboratory for Marine Biology and Biotechnology, Qingdao National Laboratory for Marine Science and TechnologyQingdao, China

**Keywords:** marine bacteria, alginate lyase, cold-adapted enzyme, salt-activated, polysaccharide lyase family 7

## Abstract

Marine bacterial alginate lyases play a role in marine alginate degradation and carbon cycling. Although a large number of alginate lyases have been characterized, reports on alginate lyases with special characteristics are still rather less. Here, a gene *alyPM* encoding an alginate lyase of polysaccharide lyase family 7 (PL7) was cloned from marine *Pseudoalteromonas* sp. SM0524 and expressed in *Escherichia coli.* AlyPM shows 41% sequence identity to characterized alginate lyases, indicating that AlyPM is a new PL7 enzyme. The optimal pH for AlyPM activity was 8.5. AlyPM showed the highest activity at 30°C and remained 19% of the highest activity at 5°C. AlyPM was unstable at temperatures above 30°C and had a low *T*_m_ of 37°C. These data indicate that AlyPM is a cold-adapted enzyme. Moreover, AlyPM is a salt-activated enzyme. AlyPM activity in 0.5–1.2 M NaCl was sixfolds higher than that in 0 M NaCl, probably caused by a significant increase in substrate affinity, because the *K*_m_ of AlyPM in 0.5 M NaCl decreased more than 20-folds than that in 0 M NaCl. AlyPM preferably degraded polymannuronate and mainly released dimers and trimers. These data indicate that AlyPM is a new PL7 endo-alginate lyase with special characteristics.

## Introduction

Alginates are mainly produced by brown algae in the ocean, which are an important component of marine organic carbon. Alginates are liner unbranched copolymer consisting of β-D-mannuronate (M) and its C5 epimer, α-L-guluronate (G), arranged in block structures such as homopolymeric G block, M block, and heteropolymeric MG (GM) blocks (Gacesa, 1988). The specific conformation of the guluronic acid region allows preference to binding divalent cations and leading to better gelling capability and gel strength (Braccini et al., 1999). Therefore, the M/G ratio, sequence and G-block length are critical factors affecting the physical properties of alginate and its resultant hydrogels (Braccini and Perez, 2001). The alginate composition, sequence and molecular weight vary with the sources that alginates are extracted from Haug et al. (1959) and Tonnesen and Karlsen (2002). Determination of the fine structure of alginate helps us to produce and use alginate molecules with defined properties optimally suited for a given application (Aarstad et al., 2012; Lee and Mooney, 2012).

Alginate degradation, which is mainly performed by alginate lyases, is a part of marine carbon cycling. Alginate lyases have been reported from marine algae, mollusks, fungi, bacteria, bacteriophages, and viruses. Alginate lyases catalyze the degradation of alginate by a β-elimination of the 4-*O*-glycosyl bond to form a double bond between C-4 and C-5, producing 4-deoxy-L-erythro-hex-4-ene pyranosyluronate at the non-reducing end of the resulting oligosaccharides (Preiss and Ashwell, 1962). Alginate lyases are classified into three types based on their substrate specificities, the first type is specific toward G blocks (EC 4.2.2.11), the second type specific toward M blocks (EC 4.2.2.3), and the third type is bifunctional for G and M blocks (Wong et al., 2000). While a large number of alginate lyases have been characterized, reports on alginate lyases with special characteristics are still rather less. Some cold-adapted alginate lyases have been found to be secreted by bacteria from the Arctic (Dong et al., 2012) and other sea (Xiao et al., 2006; Li et al., 2015). A thermophilic alginate lyase (Inoue et al., 2016), a high-alkaline alginate lyase (Kobayashi et al., 2009) and several salt-activiated alginate lyases (Horikoshi, 1982; Lange et al., 1989; Brown and Preston, 1991; Kitamikado et al., 1992; Xiao et al., 2006; Uchimura et al., 2010) have also been reported.

Based on their amino acid sequences, alginate lyases are classified into seven polysaccharide lyase (PL) families (PL5, 6, 7, 14, 15, 17, 18) in the Carbohydrate-Active enZYmes (CAZy) database^1^ (Cantarel et al., 2009). Alginate lyases from family PL7 have been widely studied, and the crystal structures of six PL7 alginate lyases have been solved. Structural analysis shows that these lyases share a common β-sandwich fold consisting of two β-sheets, in which, conserved amino acid residues compose a deep active cleft that is covered by two flexible lid loops (Yamasaki et al., 2004, 2005; Osawa et al., 2005; Thomas et al., 2013). There are both endo-lytic and exo-lytic alginate lyases in this family. The substrate specificities of PL7 alginate lyases are also diverse, including G-specific, M-specific, and bifunctional enzymes.

*Pseudoalteromonas* sp. SM0524, isolated from rotten kelp collected from the seashore of Yantai, China, is a good producer of alginate lyases (Li et al., 2011). Aly-SJ02 produced by *Pseudoalteromonas* sp. SM0524 is a PL18 bifunctional alginate lyase that mainly releases dimers and trimers from alginate (Li et al., 2011). The N-terminal extension of aly-SJ02 plays a role in the correct folding of the catalytic domain (Dong et al., 2014). In this article, we characterized another alginate lyase, AlyPM, from *Pseudoalteromonas* sp. SM0524. The result showed that AlyPM was a new cold-adapted PL7 alginate lyase with a significant preference toward polyM. Moreover, AlyPM was shown to be a salt-activated endo-enzyme, which mainly released dimers and trimers from sodium alginate and polyM. These special characteristics of AlyPM indicate that AlyPM is well adapted to marine environment and may play a role in marine alginate degradation and carbon cycling.

## Materials and Methods

### Materials and Strains

Sodium alginate from brown algae (viscosity: 2% solution at 25°C, ~250) was purchased from Sigma. PolyM and PolyG (purity: about 95%) were kindly provided by Professor Wengong Yu in Ocean University of China. Glutathione was obtained from Sigma. Glutathione Sepharose 4B was purchased from GE Healthcare. Restriction enzymes were purchased from Thermo. Human Thrombin was purchased from Novagen.

Marine bacterium *Pseudoalteromonas* sp. SM0524 was previously isolated from rotten kelp which was collected from a kelp culture field at the seashore of Yantai, China (Li et al., 2011). This strain was grown on marine broth 2216 (Difco) at 15°C. *Escherichia coli* DH5α was used for plasmid construction, and *E. coli* BL21 (DE3) was used as the host for gene expression. These strains were grown in Luria-Bertani (LB) broth or on LB broth agar (LB broth supplemented with 1.5% agar) containing 100 μg/mL ampicillin.

### Gene Cloning, Expression, and Purification of AlyPM

Two degenerate primers were designed according to two conserved sequences of 30 kDa alginate lyases, SN1 (TGGCGNCAYGARTAYAARGT) according to the sequence WRHEYKV and SC1 (TANGCNCCNGCYTTRAARTA) according to the sequence YFKAGAY. A DNA fragment was amplified from the genomic DNA of *Pseudoalteromonas* sp. SM0524 by gradient PCR (annealing temperature from 44 to 56°C) with primers SN1 and SC1. Then, three specific primers were designed based on the 5′ terminal sequence, and three based on the 3′ terminal sequence of the amplified DNA fragment. Two general primers containing the protein initiation codon and the stop codon, respectively, were also designed. Thermal asymmetric interlaced (TAIL) PCRs (Liu and Whittier, 1995) were then carried out to amplify the neighboring sequences of the DNA fragment. As a result, a upstream sequence and a downstream sequence of the DNA fragment were amplified from the genomic DNA of *Pseudoalteromonas* sp. SM0524 and sequenced. Through assembly, a DNA sequence containing a 1,089 bp ORF that encodes gene *alyPM* was obtained. After verification by PCR-amplification and sequencing, the sequence of gene *alyPM* was submitted to the GenBank database under the accession number EU548076. The signal peptide of AlyPM was predicted by SignalP 3.0 server (Bendtsen et al., 2004) and the domains of AlyPM were predicted by the search in NCBI Conserved Domain Database (CDD^2^) (Marchler-Bauer et al., 2011). Multiple amino acid sequence alignment of the deduced catalytic domain was done by Clustal X 1.83 program with other characterized alginate lyases in family PL7 (Thompson et al., 1997).

The *alyPM* gene without the signal peptide sequence was cloned into the pGEX-4T-1 vector, which was then transferred into *E. coli* BL21 (DE3). The recombinant *E. coli* BL21 (DE3) cells harboring the constructed plasmid were cultured on a LB medium containing 100 μg/mL ampicilin to OD_600_ of 0.8–1.0 at 37°C, and then cultivated in a shaking incubator at 100 rpm for 24 h at 15°C under the induction of 0.2 mM isopropyl-β-D-thiogalactopyranoside (IPTG). The cells were harvested and ultrasonically disrupted in 1 × GST binding buffer (5 mM phosphate buffer containing 137 mM NaCl, pH 7.3). After centrifugation at 12,000 *g*, 4°C for 15 min, the clear cell extract solution containing the GST-fused AlyPM was loaded on a Glutathione Sepharose 4B column equilibrated with 1 × GST binding buffer. The GST-tag of the fused AlyPM was cut off by Human thrombin at 15°C for 8 h. The released AlyPM was washed with 1 × GST binding buffer and then was stored at -20°C for further use. The purity of AlyPM was detected by sodium dodecyl sulfate polyacrylamide gel electrophoresis (SDS-PAGE) with the method of Laemmli (Laemmli, 1970). Protein concentration was determined by the bicinchoninic acid (BCA) method using a BCA protein assay kit (Thermo, USA) with bovine serum albumin as the standard.

### Enzyme Assay

Alginate lyase activity was determined by monitoring the increase of absorbance at 235 nm (*A_235_*) caused by production of unsaturated uronic acids as the lyase cleaves glycosidic bonds in the polymer chain (Gacesa, 1992). Unless otherwise noted, the activity was measured at 30°C for 30 min in a mixture of 80 μl buffer (50 mM Tris-HCl, pH 8.0), 100 μl alginate substrate (5 mg/ml), and 20 μl enzyme extract. After incubation, the mixture was boiled for 5 min to terminate the reaction. One unit of enzyme activity was defined as the amount of enzyme that increased *A_235_* by 0.1 per min.

### Enzyme Characterization

Unless otherwise noted, sodium alginate was used as the substrate in enzyme assays for AlyPM characterization. To determine the optimum temperature for AlyPM catalysis, the alginate lyase activity of AlyPM was measured at 5–45°C in 5°C intervals. The optimum pH for AlyPM catalysis was measured in different buffers ranging from pH 5.0 to 11.0. The buffers used were 50 mM phosphate buffer (pH 5.0–7.0), 50 mM Tris-HCl (pH 7.0–9.5), and 50 mM NaHCO_3_-NaOH buffer (pH 9.5–11.0). The thermostability of AlyPM was determined by measuring the residual activity after incubating the enzyme at 5–50°C for 15 min. To analyze the effects of metal ions on the activity of AlyPM, different chemicals were added to the reaction mixture at a final concentration of 2 or 10 mM, respectively. NaCl of different concentrations in the range of 0–2 M was added to the reaction mixture to investigate the effect of NaCl on the activity of AlyPM. Kinetic parameters of AlyPM in 0 and 0.5 M NaCl were determined at 30°C by non-linear analysis based on the initial rates determined from 0.3 to 18 mg/ml of substrates and analyzed by the Michaelis–Menten equation using the Origin8 Pro SR4 software. Substrate specificity of AlyPM was tested by measuring its activities toward polyM, polyG, and sodium alginate. Oligomers released from polyM, polyG, and sodium alginate by AlyPM were analyzed by thin layer chromatography (TLC) and the solvent system was 1-butanol/acetic acid/water (4:6:1, v/v). The products were visualized by heating TLC plates at 90°C for 15 min after spraying with 10% (v/v) sulfuric acid in ethanol. Standard alginate di-, tri-, and tetrasaccharides, with molecular mass of 351, 527, and 703, respectively, were prepared as described previously (Li et al., 2011).

### Circular Dichroism Spectra

AlyPM of 0.20 mg/ml in 20 mM Tris-HCl buffer (pH 8.0) with or without 0.5 M NaCl were subjected to circular dichroism (CD) spectroscopy assays at 25°C on a J-810 spectropolarimeter (Jasco, Japan). The CD spectra were recorded from 250 to 195 nm at a scan speed of 200 nm/min with a path length of 0.1 cm. Thermal unfolding (*T_m_*) value of AlyPM was determined on the same CD spectrophotometer equipped with a Peltier type temperature controller with a heating rate of 1°C/min. The concentration of AlyPM was 0.20 mg/ml in 20 mM Tris-HCl buffer (pH 8.0). Spectra were recorded from 250 to 184 nm at 0.2 nm resolution. Data were collected from 20 to 70°C in 1°C intervals. CD signals at 222 nm were used for analysis of the unfolding curve.

## Results

### Sequence Analysis of AlyPM

**FIGURE 1:**
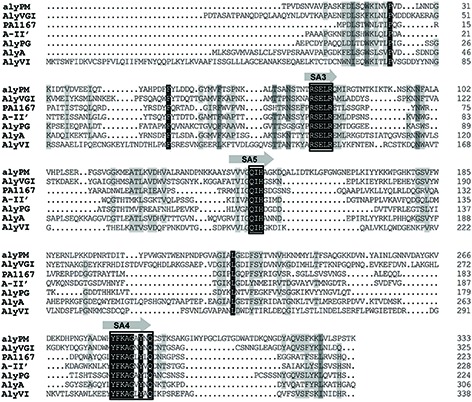
**Amino acid alignment of AlyPM to other characterized PL7 alginate lyases.** AlyPM (EU548076) from *Pseudoalteromonas* sp. SM0524 in this study; AlyVGI (AAF22512), an alginate lyase from *Vibrio halioticoli* IAM14596T; PA1167 (AAG04556), an alginate lyase from *Pseudomonas aeruginosa* PAO1; A1-II’ (BAD16656), an alginate lyase from *Sphingomonas* sp. A1; AlyPG (BAA83339), an alginate lyase from *Corynebacterium* sp. ALY-1; AlyA (AAA25049), an alginate lyase from *Klebsiella pneumoniae* SUBSP. AEROGENES; AlyVI (AAP45155), an alginate lyase from *Vibrio* sp. QY101. Identical and similar amino acid residues among the alginate lyases are shaded in black. Conserved regions are boxed.

The open reading frame of gene *alyPM* amplified from the genome of *Pseudoalteromonas* sp. SM0524 is 1,089 bp in length and encodes an alginate lyase contaning 362 amino acid residues. According to the results of signal peptide prediction by SignalP 3.0 and searching in NCBI CDD, AlyPM is a single-domain PL7 enzyme containing a 28-residue signal peptide (Met1-Ser28). Among the characterized alginate lyases, AlyPM had the highest identity (41%) to AlyVGI from *Vibrio halioticoli* IAM 14596T (Sugimura et al., 2000). Alginate lyases in family PL7 contain three highly conserved motifs, SA3 (RXEXR), SA4 (YXKAGXYXQ), and SA5 (QXH), which compose the active center and are crucial for substrate recognition and catalysis (Yamasaki et al., 2004; Kawamoto et al., 2006). Sequence alignment of AlyPM with AlyVGI and other PL7 alginate lyases that have crystal structures indicates that these conserved motifs in AlyPM are RSELR (SA3), YFKAGAYNQ (SA4), and QIH (SA5) (**Figure 1**).

### Expression and Characterization of AlyPM

**FIGURE 2:**
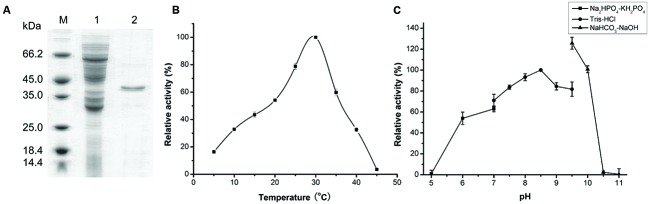
**Sodium dodecyl sulfate polyacrylamide gel electrophoresis (SDS-PAGE) analysis of purified AlyPM alginate lyase and effect of temperature and pH on AlyPM activity. (A)** SDS-PAGE analysis of purified AlyPM. Lane 1, Whole-cell protein extracts of transformed *E. coli* BL21 (DE3) cells; lane 2, purified AlyPM; lane M, protein mass markers. The proteins were subjected to electrophoresis on 12.5% acrylamide gel and stained with Coomassie Brilliant Blue R-250. **(B)** Effect of temperature on AlyPM activity. The assay was conducted at pH 8.0 in Tris-HCl buffer. Activity at 30°C was taken as 100%. **(C)** Effect of pH on AlyPM. Reactions were conducted at 30°C for 30 min in the following 50 mM buffers; PBS (closed squares), Tris-HCl (closed circles), and NaHCO_3_-NaOH (closed triangles). Activity at pH 8.5 in Tris-HCl was taken as 100%.

AlyPM without the signal peptide was expressed in *E. coli* BL21 (DE3) as a GST-fused protein, which was then bound to a Glutathione Sepharose 4B column. The recombinant AlyPM was cut off from the GST-fused protein bound to the column. SDS-PAGE analysis showed that the purified AlyPM displayed an apparent molecular weight of approximately 40 kDa, slight larger than the theoretical value (37.1 kDa) deduced from the predicted amino acid sequence (**Figure 2A**).

With sodium alginate as substrate, AlyPM showed the highest activity at 30°C (**Figure 2B**). In 50 mM Tris-HCl buffer, AlyPM had the highest activity at pH 8.5, and retained more than 70% of its highest activity between pH 7.0 and 9.5 (**Figure 2C**). AlyPM showed different activities in different buffers at the same pH, suggesting that buffer ions affect its activity. Thus, the effect of metal ions on the enzyme activity of AlyPM was measured (**Table 1**). Ni^2+^ at 2 and 10 mM both inhibited the enzyme activity by about 50%. Cu^2+^ and Co^2+^ increased the enzyme activity at 2 mM but inhibited it at 10 mM. The other metal ions, such as Ba^2+^, Ca^2+^, Mg^2+^, and Mn^2+^, had no or a little enhancing effect on the enzyme activity (**Table 1**).

**Table 1:** Effect of metal ions on AlyPM activity.

Metal ions	Relative activity (%)	
	2 mM	10 m M
None	100	100
BaCl	130.1 ± 1.4	110.1 ± 4.3
CaCl	100.6 ± 2.6	129.6 ± 1.3
CoCl	148.5 ± 12.3	73.6 ± 5.7
CuCl	133.5 ± 7.7	78.0 ± 5.0
MgCl	145.9 ± 10.8	122.9 ± 8.2
MnCl	125.0 ± 4.6	111.8 ± 4.43
NiCl	46.1 ± 6.2	50.1 ± 2.5

*^a^The activity of AlyPM in 20 mM Tris buffer (pH 8.0) without metals is taken as 100%.*

### Cold-Adapted Characters of AlyPM

**FIGURE 3:**
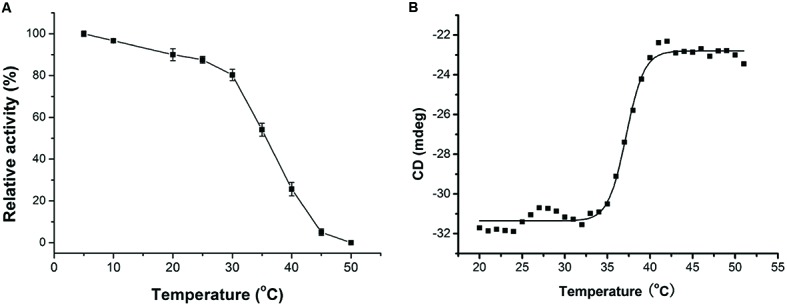
**Thermostability and melting temperature (*T_m_*) of AlyPM. (A)** Thermostability of AlyPM. The enzyme was incubated at 5 to 50°C for 15 min. The remaining activity was measured under optimal conditions. **(B)** *T_m_* valve of AlyPM. Data were collected from 20 to 70°C in 1°C intervals on a Jasco V810 Circular dichroism (CD) spectrophotometer. CD signals at 222 nm were used for analysis of the unfolding curve.

Cold-adapted enzymes usually have lower optimum temperature, higher activity at low temperatures, and lower thermostability compared to their mesophilic homologs (Lonhienne et al., 2000; Feller and Gerday, 2003). As shown in **Figure 2B**, AlyPM had a low optimal temperature of 30°C, and remained 19% of the highest activity at 5°C, and no detectable activity at 45°C. This result suggests that AlyPM has cold-adapted characters. We further investigated the thermostability of AlyPM. As shown in **Figure 3A**, AlyPM was rapidly inactivated at temperatures above 30°C, and approximately 80% of its activity was lost after incubation at 40°C for 15 min. Moreover, the apparent melting temperature (*T_m_*) of AlyPM determined by CD was only 37°C (**Figure 3B**). These results indicate that the thermostability of AlyPM is quite low, further supporting that AlyPM is a cold-adapted enzyme.

### Salt-Activation of AlyPM

**FIGURE 4:**
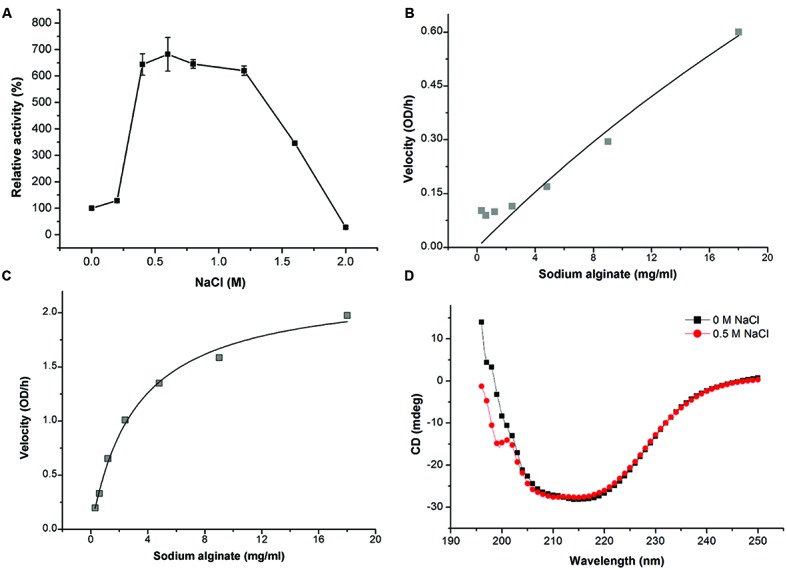
**Effect of NaCl on AlyPM. (A)** Effect of NaCl concentration on AlyPM activity. The activity was measured at 30°C for 30 min in 50 mM Tris-HCl buffer with the indicated NaCl concentrations. The activity in 0 M NaCl was taken as 100%. **(B,C)** Non-linear fit curves for the hydrolysis of sodium alginate by AlyPM in 0 M NaCl **(B)** and 0.5 M NaCl **(C)**. **(D)** CD spectra of AlyPM in 0 and 0.5 M NaCl.

Because AlyPM is from a marine bacterium, we studied the effect of NaCl of different concentrations on the activity of AlyPM. Surprisingly, we found that the activity of AlyPM was significantly enhanced approximately sixfolds by 0.5–1.2 M NaCl (**Figure 4A**). This result indicates that AlyPM is a salt-activated enzyme (Kitamikado et al., 1992; Wong et al., 2000; Kobayashi et al., 2009).

To investigate whether the enhancement of AlyPM activity by NaCl was attributed to an increase in substrate affinity, we compared the *K*_m_ values of AlyPM toward sodium alginate under 0 and 0.5 M NaCl. The *K*_m_ values were determined by non-linear analysis based on the initial rates determined between 0.3 and 18 mg/ml of sodium alginate because 18 mg/ml was nearly the biggest solubility of sodium alginate in 50 mM Tris buffer. The result showed that the *K*_m_ value of AlyPM in 0.5 M NaCl (3.15 mg/ml) decreased by more than 20-folds compared to that in 0 M NaCl (74.39 mg/ml) (**Figures 4B,C**). This indicated that the substrate affinity of AlyPM in 0.5 M NaCl was significantly increased, which probably resulted in the enhancement of AlyPM activity. We also compared the CD spectra of AlyPM in 0 M NaCl and 0.5 M NaCl, which showed no detectable changes between them (**Figure 4D**). This suggested that the increase in the substrate affinity of AlyPM in 0.5 M NaCl was not caused by structural changes.

### Substrate Specificity and Action Mode of AlyPM

**FIGURE 5:**
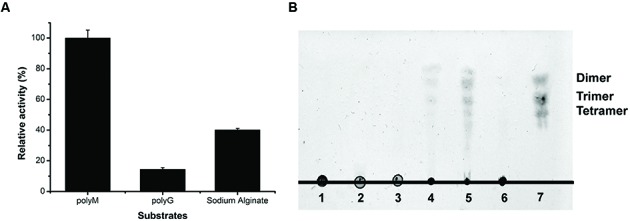
**Substrate preference and action mode of AlyPM. (A)** Substrate specificity of AlyPM evaluated with sodium alginate, polyM, and polyG. **(B)** TLC analysis of the oligomers released from polyM, polyG, and sodium alginate by AlyPM. A 500 μL reaction mixture containing 1 μg AlyPM and 2.5 mg polyM, polyG, or sodium alginate was incubated at 30°C for 2 h. Lane 1, sodium alginate; Lane 2, polyM; Lane 3, polyG; Lane 4, reaction products generated from sodium alginate. Lane 5, reaction products generated from polyM; Lane 6, reaction products generated from polyG. Lane 7, standard alginate oligomers.

To determine the substrate specificity of AlyPM, the enzyme activities toward sodium alginate, polymannuronate (polyM), and polyguluronate (polyG) were measured. As shown in **Figure 5A**, AlyPM showed the highest activity toward polyM, and only a little activity toward polyG, indicating that AlyPM had a preference to polyM than to polyG. The oligosaccharides released from polyM, polyG, and sodium alginate through the action of AlyPM were analyzed by TLC (**Figure 5B**). The maximum oligosaccharide production generated by AlyPM was from polyM and no oligosaccharide was detected from polyG on the TLC plates, which agreed with the substrate specificity of AlyPM. Products from sodium alginate and polyM generated by AlyPM were mainly dimers and trimers, and also some tetramers and even larger oligosaccharides. Thus, AlyPM mainly acts on substrate endolytically. In addition, some products lower than dimers were also shown on the TLC plates. It remains to be further determined whether AlyPM has exotype activity.

## Discussion

In this study, a gene encoding an alginate lyase AlyPM from marine bacterium *Pseudoalteromonas* sp. SM0524 was cloned. Sequence analysis showed that AlyPM belongs to the PL7 family. The PL7 family has the most alginate lyases among the seven PL families containing alginate lyases. There are now more than 535 PL7 alginate lyase sequences in the CAZy database^3^ (Cantarel et al., 2009). Moreover, various alginate lyases with different substrate specificity and characters have been characterized [for details see review of Zhu and Yin (2015)]. Among the characterized alginate lyases, AlyPM has the highest identity (41%) to AlyVGI from *V. halioticoli* IAM14596T (Sugimura et al., 2000), which indicates that AlyPM is a new member of the PL7 family.

A few cold-adapted alginate lyases have been characterized. Consistent with other cold-adapted enzymes investigated, the cold-adapted alginate lyases studied usually have lower temperature optimum, higher catalytic activities at low temperatures and lower thermostability than their mesophilic homologs (Xiao et al., 2006; Dong et al., 2012; Li et al., 2015). For an example, mesophilic alginate lyases usually have optimal temperatures around 50°C and generally stable at temperatures lower than 50°C (Kim et al., 2012; Park et al., 2012; Dou et al., 2013), whereas cold-adapted alginate lyases usually have optimal temperatures less than 35°C and generally unstable at temperatures higher than 30°C (Xiao et al., 2006; Li et al., 2015). We studied the effect of temperature on the activity and stability of AlyPM. The result showed that AlyPM had a low optimal temperature (30°C) for activity, and still remained 19% of the highest activity at 5°C. Moreover, AlyPM was quite thermolabile. It was unstable at temperatures beyond 30°C, and had a very low *T*_m_ (37°C). These results indicate that AlyPM is a cold-adapted alginate lyase. *Pseudoalteromonas* sp. SM0524 that secretes AlyPM was isolated from the seashore of the Yellow Sea where the temperature of seawater is usually less than 28°C (Fan and Huang, 2008; Payne et al., 2012). Therefore, the cold-adapted character of AlyPM reflects its adaptation to the marine environment where it functions.

Several salt-activated alginate lyases have been reported, whose activities can be increased by cations such as sodium and calcium (Horikoshi, 1982; Lange et al., 1989; Brown and Preston, 1991; Kitamikado et al., 1992; Wong et al., 2000; Xiao et al., 2006; Uchimura et al., 2010). Among them, an alginate lyase from *V. harveyi* AL-128 (Kitamikado et al., 1992) and A9mT from *Vibrio* sp. JAM-A9m (Uchimura et al., 2010) are the most salt-activated, and their activities are both increased by 24 times at 1 and 0.4 M NaCl, respectively. However, the salt-activated mechanism of alginate lyases is still unclear. Cations are thought to decrease the surface density of the substrate charge, weakening the ionic interactions between alginate and the enzyme (Favorov et al., 1979). In this study, we found that AlyPM is a salt-activated alginate lyase, whose activity could be increased by six times at 0.5–1.2 M NaCl. Further analysis indicated that AlyPM has a much higher affinity to substrate in 0.5 M NaCl than in 0 M NaCl, which probably leads to the increase of its activity in 0.5 M NaCl. The salt-activated character of AlyPM reflects its adaptation to the salty seawater environment.

A large amount of alginates are annually produced by algae in the sea. Various alginate lyases secreted by marine bacteria are actively involved in marine alginate degradation, and therefore, play a role in marine carbon cycling. In this study, AlyPM secreted by marine bacterium *Pseudoalteromonas* sp. SM0524 was characterized as a new PL7 alginate lyase. AlyPM is cold-adapted and salt-activated, and can hydrolyze alginate to produce oligomers. These characteristics indicate that AlyPM is well adapted to marine environment and may play a role in marine alginate degradation and carbon cycling. In additon, the cold-adapted and salt-activated alyPM may have potentials in some biotechnology areas and industries, which merits further study.

## Author Contributions

SD, FX, and FD performed all experiments. X-LC and B-BX directed the experiments. X-LC and SD wrote the manuscript. X-YZ and P-YL analyzed the data. B-CZ and Y-ZZ designed the research.

## Conflict of Interest Statement

The authors declare that the research was conducted in the absence of any commercial or financial relationships that could be construed as a potential conflict of interest.

## Footnotes

1 http://www.cazy.org/fam/acc\_PL.html

2 http://www.ncbi.nlm.nih.gov/Structure/cdd/cdd.shtml

3 http://www.cazy.org/fam/acc_PL.html
